# Aromaticity switching by quantum tunnelling

**DOI:** 10.1039/d5sc05717e

**Published:** 2025-10-07

**Authors:** Sindy Julieth Rodríguez-Sotelo, Juan Julian Santoyo-Flores, Katarzyna Młodzikowska-Pieńko, Renana Gershoni Poranne, Sebastian Kozuch

**Affiliations:** a Ben-Gurion University of the Negev Beer-Sheva 841051 Israel sindyjuliethr@gmail.com; b Schulich Faculty of Chemistry, Technion-Israel Institute of Technology Technion City Haifa 32000 Israel

## Abstract

Antiaromatic π-conjugated systems provide a powerful framework for understanding ultrafast molecular rearrangements driven by quantum tunnelling over their degenerate double-well potential surfaces. Here, we explore with computational tools the π-bond-shifting automerization in the antiaromatic dinaphtho[2,1-*a* : 1,2-*f*]pentalene (1), dinaphtho[1,2-*a* : 2,1-*f*]pentalene (2), and a series of substituted derivatives. These molecules exhibit a distinctive feature: local aromatic and antiaromatic rings can interconvert their aromaticity character even close to the absolute zero *via* unusually fast carbon tunnelling. If these systems can be prepared in a coherent regime, the quantum superposition between the original states would delocalise their nuclear wavefunctions in a state that we describe as a “Schrödinger's aromaticity cat.”

## Abstract 2

Carbon tunnelling in pentalene-based polycyclic molecules enables ultrafast π-bond rearrangements switching local aromaticity/antiaromaticity, or leading to a quantum “Schrödinger's aromaticity cat” in a coherent tunnelling regime.

## Graphical abstract

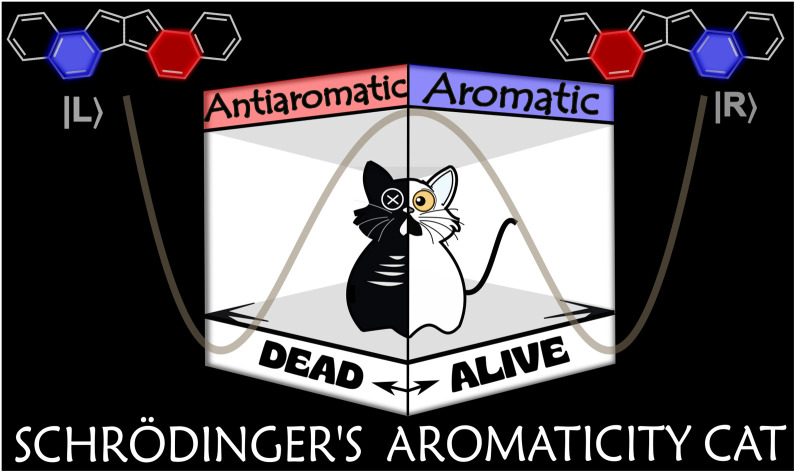

## Introduction

Symmetrical isothermal automerizations characterised by degenerate double-well potential energy surfaces (**DWP**) represent a fascinating class of systems in which a particle—be it an electron, an atom, or even a whole molecule—is confined between two equivalent minima separated by a finite energy barrier.^1,2^ These systems provide a natural setting for the study of the quantum tunnelling (**QT**) effect, if the height and width of the barrier, as well as the tunnelling reduced mass, are low enough.^3,4^ This is especially relevant in cryogenic conditions, where thermal activation is suppressed.^5^

Moreover, the tunnelling can occur in two flavours. In the coherent regime, a delocalisation between the left (|L〉) and right (|R〉) wells occurs, resulting in a measurable energy level splitting. In the decoherent regime, the system is localised in |L〉 or |R〉, but can swiftly move back and forth between them. It is possible to say that the first is a physical process, whereas the second is of a more chemical nature. Of note, interactions with a bath or simply with other molecules can make the coherent tunnelling decohere, as explained elsewhere.^4,6,7^ These processes have been essential in explaining phenomena such as proton transfer in biological systems,^8^ quantum dynamics in functional materials,^9^ and molecular switching in quantum devices.^10^

Antiaromatic compounds^11^ represent particularly intriguing cases, as their π-bond shifting can lead to automerization reactions suitable for QT.^12–15^ One of the most studied antiaromatic compounds is pentalene,^12,16–18^ a bicyclic 8π-electron species that exhibits a small HOMO–LUMO energy gap^18,19^ and distinctive paratropic ring currents.^20–23^

Importantly, if the pentalene-based molecule has an appropriate symmetry, it can tunnel between the two equivalent positions on the DWP, undergoing an extremely fast π-bond shifting with a computed rate constant on the order of 10^8^ s^−1^, even near the absolute zero.^12,17^ All these features further enhance the chemical and theoretical interest in pentalene-based systems.

**Fig. 1:**
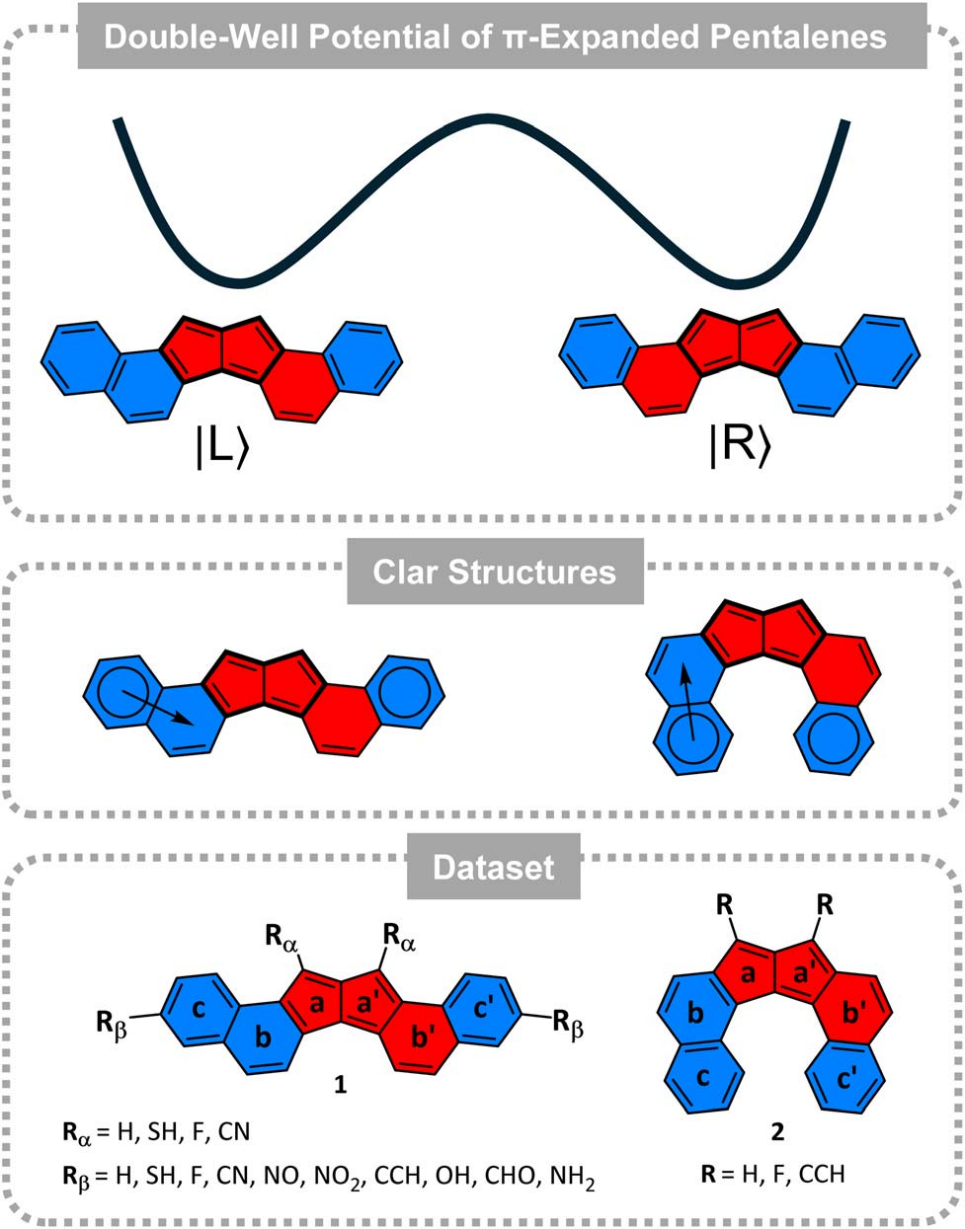
(Top) schematic depiction of the double-well potential and the two minima structures. The |L〉 and |R〉 states have the same energy but different local (anti)aromaticity. (Middle) Clar structures depicting the location of the fixed and migrating Clar sextets (the arrows indicate the migration of the Clar sextet, permitting to move the circle to the indicated ring). (Bottom) dinaphtho-[2,1-*a* : 1,2-*f*]pentalene (1) and dinaphtho-[1,2-*a* : 2,1-*f*]pentalene (2). In all panels: blue indicates aromatic/diatropic, red indicates antiaromatic/paratropic.

Herein, we studied the QT-included kinetic behaviour of dinaphtho-[2,1-*a* : 1,2-*f*]pentalene (1), dinaphtho-[1,2-*a*,2,1-*f*]pentalene (2), and several substituted derivatives. The fusion of pentalene with aromatic ring subunits is a strategy used in these systems to stabilise the reactive antiaromatic core and modulate the HOMO–LUMO energy gap to enhance the charge delocalisation and transport properties.^24–35^ Notably, some fused pentalene derivatives of this family were originally designed and experimentally realized by Konishi and co-workers.^24,35^ These pentalene-based architectures define a new class of systems that, like the parent pentalene, may rearrange by QT in a symmetric DWP, where |L〉 and |R〉 are isoenergetic species. However, in contrast to the parent case, these isomers exhibit unusual aromaticity dynamics.^24,28–33^ Tunnelling in pentalene rearranges the pattern of single and double bonds within the π-conjugated framework, but does not change the aromatic character of the individual rings or the bicyclic system. In contrast, compounds 1 and 2 have unsymmetric local aromatic behaviour (see Fig. 1), which must rearrange concurrently with the π-bond automerization. We hypothesise here that the reaction will be extremely fast due to the tunnelling effect. If so, in the localised regime rings b and b′ (see Fig. 1, bottom) will continuously undergo a quantum-driven redistribution of their local aromaticity even at deep cryogenic conditions, at a rate that would make it impossible to define their character (unless external forces break the molecular symmetry, of course).^36–38^ Moreover, if the molecule can be prepared in a coherent state, these rings will be both aromatic and antiaromatic, in a kind of metaphorical “Schrödinger's aromaticity cat”.

Furthermore, we show that it is possible to modulate the aromatic lack of symmetry, reaction rates, and energy splittings by substitution with functional groups (we considered two positions in 1 and one position in 2). With this information, we could obtain relationships between the tunnelling kinetics, electronic factors, and aromaticity measures.

## Computational methods

All molecular structures were optimised at the M06-2X/6-311+G(d) level,^39^ and their minima confirmed through frequency computations using Gaussian 16.^40^ This functional has proven to be adequate for organic reactions, while at the same time, it is fast enough for the costly tunnelling computations. Both 1 and 2 are characterized by a closed-shell ground-state configuration,^19,22^ as are all other molecules studied herein (see more details in the SI). More accurate threshold and reaction energies computations were carried out using DLPNO-CCSD(T1)/cc-pVQZ^41,42^ with TIGHTSCF and TIGHTPNO options over the DFT geometries with ORCA 6.^43,44^ All the energies presented are zero-point corrected energies (obtained with M06-2X).

The semiclassical rate constants were computed with canonical variational transition state theory (CVT),^45^ and the QT corrected values (*k*) were obtained with the small curvature tunnelling (SCT) method^46^ using Polyrate 17 (an example of a Polyrate input file is provided in the SI).^47^ To obtain more accurate rate constants, the previously obtained DLPNO-CCSD(T1) Δ*E*^‡^ and Δ*E*_r_ values were used with the ISPE double-layer method over the DFT surface.^48^ We also added the quantized reactant state tunnelling (QRST) method to include quantized vibrations at low temperatures,^49^ a small step size of 0.001 au for a high-quality potential energy surface (**PES**), and confirmed convergence of the reaction pathway for each QT computation (see an example of the PES in Fig. S9).

We initially generated a dataset of 29 derivatives by symmetrically substituting 1 and 2 at the α or β positions, including the following list of substituents: F, CN, CHO, NO, NO_2_, CCH, OH, NH_2_, and SH (the latter widely used in molecular electronics due to its strong binding affinity to gold electrodes).^50^ From this set, we retained those that fulfilled the criteria of having a closed-shell singlet electronic configuration for all stationary points. This ensures the accuracy of the single-reference electronic structure methods. Based on this, a total of 14 systems were retained for further analysis (Table 1).

**Table 1:** Heat map representation of the NICS(1)_*zz*_ values for each ring in 1 and 2. The individual entries are colour-coded with respect to their unsubstituted parent system. Blue – reduced value; red – increased value. The strength of colour is proportional to the change in NICS(1)_*zz*_ value

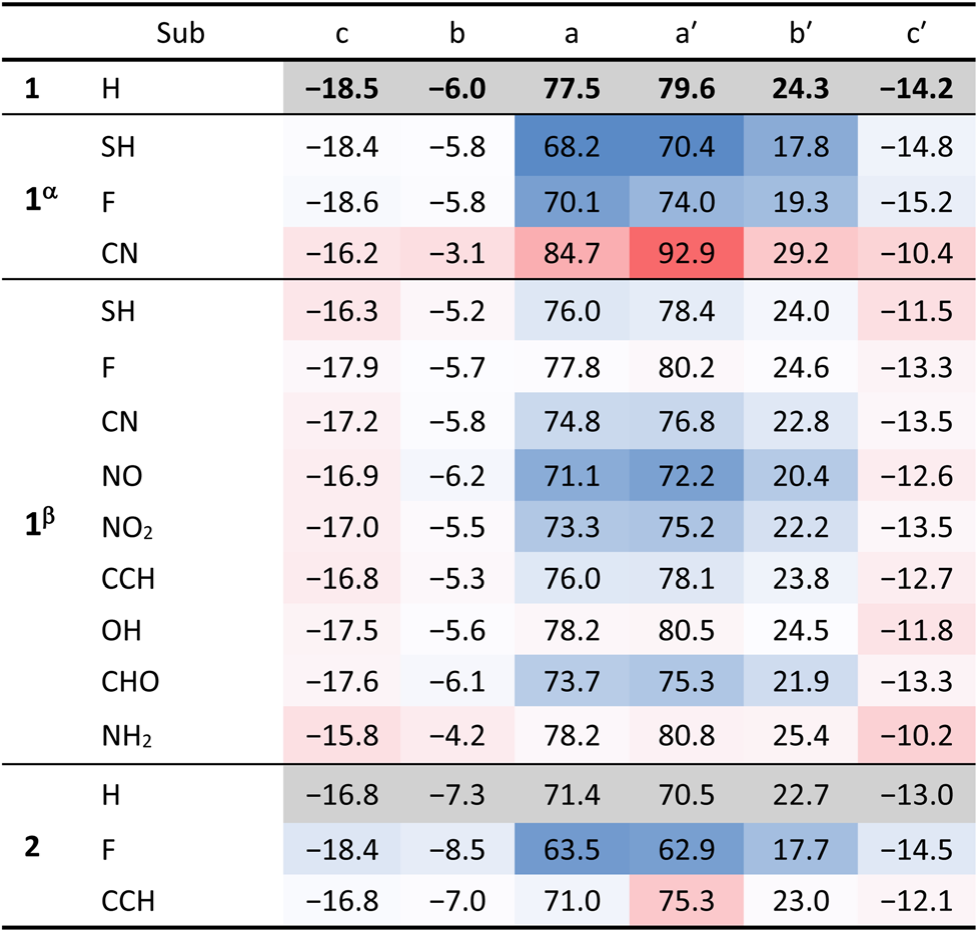

Nucleus-independent chemical shifts^51,52^ (**NICS**) were computed using the Gauge-Independent Atomic Orbital (GIAO)^53^ method at the PBE0/6-311+G(d)^54^ level of theory. To this end, we used Gaussian 16 together with Aroma 2.0 (a utility package for generating input files and parsing results of NICS calculations).^55–57^ To minimize σ-effect contaminations, we used the NICS(1)_*zz*_ metric, which reports only the *zz*-component of the chemical shift tensor and is computed at a height of 1 Å above the ring plane (for additional results calculated at 1.7 Å see SI). 2D NICS calculations were prepared and analysed using pyAroma4 at a height of 1 Å, with the same computational method described above.^58^

Current density maps were also obtained in the *Z* orientation of the magnetic field at 1 Å above the molecular plane, using the SYSMOIC program package.^59,60^ Because most of the molecules are planar, we could identify the various molecular orbitals and consider only the contribution of the π-orbitals, affording a cleaner analysis of the aromatic behaviour. Clockwise arrows are assigned to the diatropic current (aromatic), anticlockwise to the paratropic (antiaromatic).

While current density plots provide a very intuitive picture of the induced ring currents, they cannot be quantitatively compared. Therefore, we computed the net bond current strength to obtain a quantitative representation of the magnetically induced ring current density. This is obtained by integration of the induced current densities flowing through planes bisecting selected bonds. When delocalisation is present, a sizable net bond current flow is detected, whose strength can be used to compare between different parts of a molecule or between molecular systems. All net current strengths reported herein are normalised in relation to benzene (computed with M06-2X/6-311+G(d)), for which the net diatropic current strength is 11.64 nA/T.

## Results and discussion

### (Anti)aromaticity

**Fig. 2:**
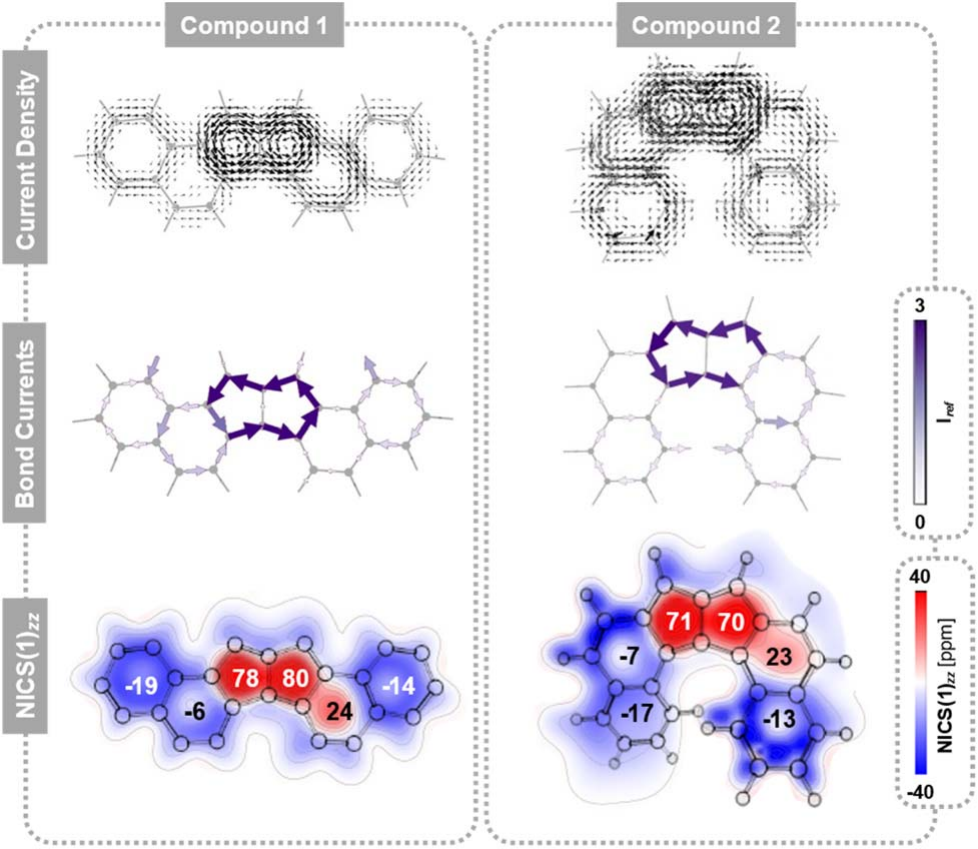
Aromaticity comparison between 1 and 2. (Top) current density plots generated with SYSMOIC at a height of 1 Å above the molecular plane under a perpendicular magnetic field (only the contribution of the π-orbitals to the induced current density is taken into account). (Middle) net bond current strengths reported relative to the bond current strength of benzene. (Bottom) 2D NICS(1)_*zz*_ plots (in ppm). The NICS(1)_*zz*_ value of each ring is denoted within it.

1 and 2 are both conjugated hexacyclic systems containing 24 π e^−^, making them formally antiaromatic. However, neither molecule displays global antiaromatic character. Instead, as shown by the current density plots, bond currents, and NICS values (Fig. 2), both isomers can be perceived as consisting of three distinct regions, which are consistent with the presence of a migrating and a fixed Clar sextet, respectively (Fig. 1):^61^ an aromatic bicyclic component (rings c and b), an antiaromatic tricyclic component (rings a, a′, and b′), and an aromatic monocyclic component (ring c′). The resulting uneven distribution of (anti)aromaticity generates an intrinsic disparity in the π-conjugated topology, which plays a central role in the DWP.

These local behaviours are reminiscent in shape of their parent components (*i.e.*, naphthalene, pentalene, benzene) but vary in their strength. In particular, we observe that the local paratropic currents are stronger than in the parent pentalene (NICS(1)_*zz*_ = 54 ppm for pentalene, and ranges between 70 and 80 ppm for rings a and a′ in 1 and 2), and are no longer identical. The retention of strong local paratropic currents in the five-membered rings is characteristic of π-extended pentalene derivatives.^62,63^ However, the change in magnitude upon fusion of aromatic rings is highly dependent on the type and direction of annulation, as shown for pentalene and other antiaromatic cores, such as *s*-indacene.^28,64,65^ In contrast, the local aromaticity in the six-membered rings is substantially attenuated (NICS(1)_*zz*_ ranging between −13 and −19 ppm for rings c and c′ in 1 and 2), a common consequence of annelation to antiaromatic cores. Rings b and b′ show weak behaviour (NICS(1)_*zz*_ ≈ −7 and 23 ppm, for b and b′, respectively). For comparison, NICS(1)_*zz*_ for benzene and cyclobutadiene at the same level of theory are −30 and 58 ppm, respectively.

The lack of symmetry is not surprising and has been previously rationalised with molecular orbital theory and symmetry considerations.^66^ It has also been interpreted as the presence of multiple local and semi-local induced current circuits. Both Konishi *et al.*^24^ and Baranac-Stojanovic^25,33^ have proposed that these molecules sustain a semi-local paratropic current that encompasses the tetracyclic b-a-a′-b′ component (in addition to other circuits). This interpretation aligns with our own calculations, while explaining the reduced aromaticity of ring b.

### Localised tunnelling

We now turn our attention to the reaction rates at deep cryogenic conditions, that is, ground state QT of 1 and 2. The degenerate rearrangement of these species by π-bond shifting resembles the reaction of the parent pentalene.^12,17^ But as can be seen in Fig. 1, in 1 and 2, the automerisation partly includes the attached six-membered rings, including their electronic characteristics (see geometries in the SI).

1 has a slightly lower threshold energy than 2 (23 *vs.* 28 kJ mol^−1^, respectively). But contrary to semi-classical kinetics, the mass of the moving particles and especially the barrier width can also affect the QT reactivity according to eqn (1):^4^1
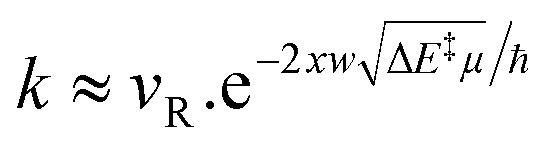
with *k* being the ground state QT rate constant, *ν*_R_ the frequency of the reactant's normal mode in the direction of the reaction, *w* the barrier width, Δ*E*^‡^ the threshold energy, *μ* the reduced moving mass, *ħ* the reduced Planck constant, and *x* a factor corresponding to the barrier shape (for example 
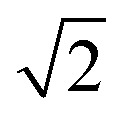
 for a rectangular barrier, and 
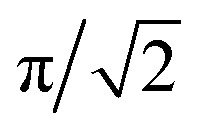
 for a parabolic barrier). Therefore, there will not be a perfect correlation between *k* and Δ*E*^‡^.

Both compounds have extremely fast reaction rates, with deep tunnelling frequencies for the back-and-forth reaction on the order of a nanosecond. The ground state QT automerization rate constant of 1 is an order of magnitude faster than that of 2, 2 × 10^9^*vs.* 2 × 10^8^ s^−1^, respectively.

At these rates, it would be difficult to experimentally detect the molecules in one of the two geometric minima. Nuclear magnetic resonance (NMR) measurements will mistakenly see the molecules as completely symmetric (*C*_2v_ instead of the real *C*_s_) since the rearrangement is much faster than the NMR time scale,^67,68^ and therefore the peaks of almost equivalent atoms will coalesce. This also means that any NMR estimation of rings b and b′ in the free molecules will show negligible aromaticity. This is not because there is none, but because the extremely fast switching between their aromatic and antiaromatic character would display an average outcome (the time scale of IR spectroscopy is much faster, potentially permitting the resolution between frequencies of almost equivalent bonds' peaks).

**Fig. 3:**
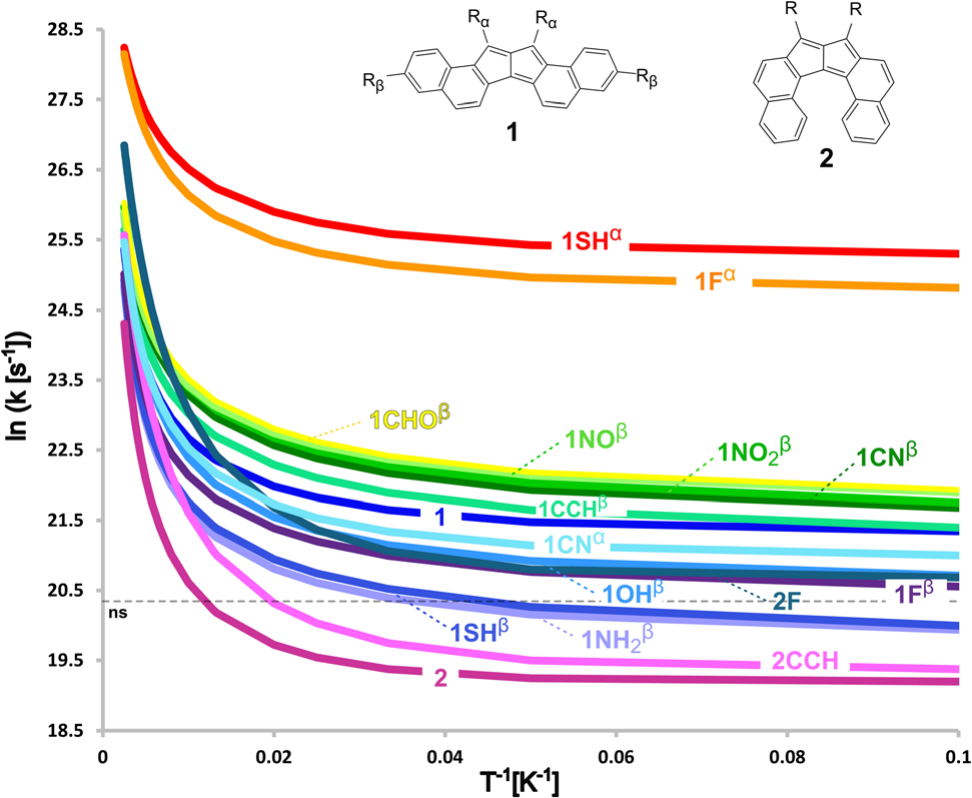
Arrhenius plot for the degenerate rearrangements of 1 and 2. The horizontal dotted line shows, as a reference, a half-life of a nanosecond.

The effect of temperature on the reaction rates is illustrated in the Arrhenius plot shown below in Fig. 3. Interestingly, the relative impact of QT is large even at room temperature, a signature of an exceptionally narrow barrier. Due to the larger influence of Δ*E*^‡^ on semiclassical kinetics compared to QT, reactions with higher threshold values have larger transmission coefficients (*κ*) values. Consequently, our calculations indicate that at 298 K the tunnelling rearrangement of 1 is a hundred times faster than considering a purely semiclassical reaction (*i.e.* without QT), while for 2 it is two hundred times faster (see SI). Evidently, even at this high temperature the reactivity is mainly driven by (thermally activated) tunnelling.^12,69^ This was already seen in the automerisation of cyclobutadiene.^14^

### Coherent tunnelling

All the previous discussion involves localised QT, where the system physically rearranges between |L〉 and |R〉. Alternatively, if these molecules can be prepared in a coherent state,^7^ we expect them to exhibit measurable splitting energies (Δ*E*_01_), which can be observed under careful spectroscopic settings. This is caused by the energy difference between the states produced by the constructive and destructive quantum superposition of the original left and right states, that is2
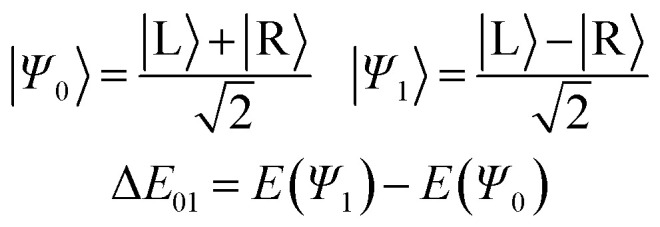


The splitting energies can be obtained from the computed ground state rate constants according to eqn (3):^4^3
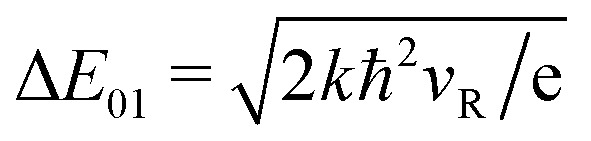


The obtained Δ*E*_01_ for 1 and 2 were 40 and 10 GHz, respectively. These values are comparable to the ammonia umbrella inversion (24 GHz), as well as other hydrogen QT reactions.^4^

For all these compounds, as long as the coherent regime is maintained, rings b and b′ will be both aromatic and antiaromatic, or neither aromatic nor antiaromatic, depending on their quantum state, |*ψ*_0_〉 or |*ψ*_1_〉 (thus the Schrödinger's aromaticity cat metaphor).

Of note, since we are reaching the maximum possible tunnelling rates in chemical reactions (on the *ν*_R_ regime of the order of 10^13^ s^−1^, see eqn (1)), we are also reaching the maximum splitting energies accessible by chemical reaction techniques (∼10^6^ MHz). Masers of higher frequencies are possible, but based on physical effects instead of chemical reactions (such as the stimulated emission between hyperfine energy levels).

### Substituents and trends

We finish this project by evaluating the influence of substitutions on the local (anti)aromatic character, and on the tunnelling rates. All the studied cases which fit the above-described requirements (symmetry and closed-shell singlet electronic structures) are presented in Fig. 1.

The variation of the NICS(1)_*zz*_ values reveals a clear modulation of the (anti)aromaticity, depending on the nature and position of the substituent (Table 1; red indicates an increase in value and blue indicates a decrease in value, relative to the same ring in the respective unsubstituted molecule).

Only three 1^α^-substituted isomers met our closed-shell transition state criteria for inclusion in this study: SH, F, and CN. The first two show a decrease in the paratropicity of rings a, a′, and b′ (a reduction ranging between 5 and 9 ppm) and a negligible change in the diatropicity of the other rings. The impact of SH on the NICS(1)_*zz*_ is larger than F, which is consistent with it being the stronger π-electron-donating group (according to Hammett and Swain–Lupton parameters).^70^ These results also align with previous investigations into substituent effects on pentalene.^71^ In contrast, the CN-substituted molecule shows a substantial increase in paratropicity in rings a, a′, and b′ (NICS(1)_*zz*_ values increasing from 5 to 13 ppm), concurrent with a slight decrease in the diatropicity of the other rings (by *ca.* 3 ppm).

When the same substituents are placed in the β position, their impact on rings a, a′, and b′ is essentially negligible, but the effect on rings c and c′ is slightly stronger. For this position, there are additional derivatives, which allow us to ascertain that the decrease in diatropicity in rings c and c′ follows the order NH_2_ > SH > OH > F, consistent with the π-electron donating ability. Interestingly, the CN group has an opposite impact when placed in the β position—it slightly decreases the paratropicity of rings a, a′, and b′, despite the greater distance from the core. Similar effects are seen with the other σ-electron withdrawing substituents, following the order NO > NO_2_ > CHO > CN > CCH, again consistent with Hammet and Swain–Lupton parameters.

For 2, we have only two derivatives. The F-substituted molecule behaves similarly to the 1^α^ isomers, which is not surprising, considering that both have the F groups located at the same position on the pentalene. The CCH-substituted molecule shows no effect on any of the rings, with the exception of ring a′, which displays an increase in paratropicity. One possible explanation for the more localised effect is the non-planarity of the 2 derivatives, which weakens the conjugation of the substituent into the larger conjugated framework.

Overall, the antiaromaticity of the pentalene core in 1 and 2 appears to be robust to a broad range of substituents. At the same time, the strength of the paratropicity is sensitive to substitution in general, as well as to the specific location of the substituent.^71^ This range of modulation is similar to *s*-indacene and other antiaromatic cores.^65^ Notably, in all cases we observe a divergent response for rings b and b′, which highlights the non-equivalent topological roles of these positions, with b′ displaying a stronger sensitivity to substituent effects and a closer electronic interaction with the pentalene core.

As with the aromaticity, even if they are all extremely fast, the rate constants also reveal some sensitivity to the electronic nature of the substituting groups, but higher sensitivity to their location. In 1, even small changes in the electronic character at position α significantly modulate the rate of rearrangement (see Table 2). For example, π-electron donor groups such as F and SH notably lower the threshold energies to approximately a quarter of their original values, increasing the rate constants by more than an order of magnitude, even considering the larger masses.

**Table 2:** ZPE included threshold energies in kJ mol^−1^, imaginary frequencies at the transition state in cm^−1^, average change of the C–C bond distances in the pentalene core as a proxy measure of the molecular displacement (in Å), ground state tunnelling rate constants in s^−1^, and splitting energies in MHz

	Sub	Δ*E*^‡^	*ν* ^‡^	|Δ*d*|	*k*	Δ*E*_01_
1	H	23.1	1946	0.109	2 × 10^9^	4 × 10^4^
1^α^	SH	5.2	853	0.088	1 × 10^11^	3 × 10^5^
	F	7.6	1016	0.090	6 × 10^10^	2 × 10^5^
	CN	23.6	2050	0.109	1 × 10^9^	4 × 10^4^
1^β^	SH	25.2	1934	0.110	5 × 10^8^	2 × 10^4^
	F	25.8	2198	0.111	8 × 10^8^	3 × 10^4^
	CN	20.1	1753	0.105	3 × 10^9^	5 × 10^4^
	NO	18.9	1612	0.103	3 × 10^9^	6 × 10^4^
	NO_2_	18.6	1650	0.104	3 × 10^9^	5 × 10^4^
	CCH	22.3	1914	0.107	2 × 10^9^	4 × 10^4^
	OH	27.0	2471	0.113	1 × 10^9^	3 × 10^4^
	CHO	19.3	1648	0.105	3 × 10^9^	6 × 10^4^
	NH_2_	29.1	2704	0.113	5 × 10^8^	2 × 10^4^
2	H	28.5	2224	0.109	2 × 10^8^	1 × 10^4^
	F	12.3	1163	0.094	1 × 10^9^	3 × 10^4^
	CCH	21.1	1946	0.104	3 × 10^8^	2 × 10^4^

In addition to the barrier height, the size of the bond-length alternation between |L〉 and |R〉 should also affect the tunnelling, as it determines the nuclear displacement and thus the QT effective mass. The transition state curvature is sometimes used as a measure of the barrier width, with larger imaginary frequencies corresponding to narrower barriers; however, as seen in Table 2, a lowering of the barrier height usually involves a lowering of the TS imaginary frequency as the PES flattens, which does not necessarily indicate narrower potentials. To have a better indication of the range of atomic movements in the automerizations, we calculated the average difference between almost equivalent C–C bond lengths at both sides of the pentalene core, which can be considered as the mean alternation, and is directly correlated to the carbon movement. As shown in Table 2, lower barrier reactions tend to come with shorter movements (*R*^2^ = 0.96), agreeing with the fact that in the limit of zero threshold energy the DWP turns into a one-well, with zero alternation. Still, the differences in the displacements are relatively small, with a maximum difference of 28%, while the threshold energies have maximum disparities of 460%. Hence, the tunnelling rates of these systems are governed mostly by their activation energies, but the structural reorganisation involved in crossing the barrier also affects it, to some extent.

The reduction in threshold height can come from two potential origins: the substituents destabilising the reactant, or stabilising the transition state (TS). As noted before, these substituents reduced the antiaromaticity of the reactants' pentalene core. In contrast, the *C*_2v_-symmetric TSs sustain much stronger global paratropic currents, and do not have any diatropic regions. Hence, we hypothesise that in these highly delocalised structures the π-electron-donating nature of these substituents stabilises the TS more than the reactant.

This is consistent with the observation that electron-withdrawing groups in position α, such as CN, slightly increase the threshold energies and lead to a moderate decrease in the rate constant. The same substituent increased the antiaromaticity of the reactant's pentalene core, and it stands to reason that the effect would be exacerbated at the strongly delocalized TS.

For the substitution at β we tested more groups, with an opposite and more moderate effect. π electron donors slightly raise the barrier and acceptors lower it, with the concomitant effect on the rates of reducing and enhancing the tunnelling efficiency, respectively. However, the changes are small because of the highly probable QT, as explained above. As can be seen in Table 2, the substitution in 2 has a similar effect to that in 1^α^, which is not surprising considering that it was done at the same positions. In essence, the tunnelling process is centralised at the pentalene core, and therefore it is logical that the α position is more sensitive to changes.

The effect of temperature on the reaction rates of all the studied systems is illustrated in the Arrhenius plot shown in Fig. 3. At 298 K, our calculations indicate that from all our systems, 1NH_2_^β^, being the one with the highest Δ*E*^‡^, reaches the highest *κ* value of almost five hundred (see SI). Even at this high temperature, the reactivity in all these systems is mainly driven by (thermally activated) tunnelling.^12,69^

For such fast reactions, all the Δ*E*_01_ were on the order of tens of GHz (Table 2). 1SH^α^ and 1F^α^ show the largest splittings of more than 10^5^ MHz (similar to malonaldehyde, a standard hydrogen QT case typically used for splitting studies).^4^

Since substituents also modulate the local (anti)aromatic character, we considered whether the tunnelling dynamics could be connected to the magnetic aromatic character (as captured by the NICS metric). Specifically, we hypothesised that the antiaromaticity of the a–a′-b′ component might correlate with threshold energies, which in turn can influence the rate of rearrangement. This led us to investigate whether a quantitative relationship could be established between NICS(1)_*zz*_ values and rate constants, a connection that could enable the predictive design of tunnelling-tuned conjugated systems.

**Fig. 4:**
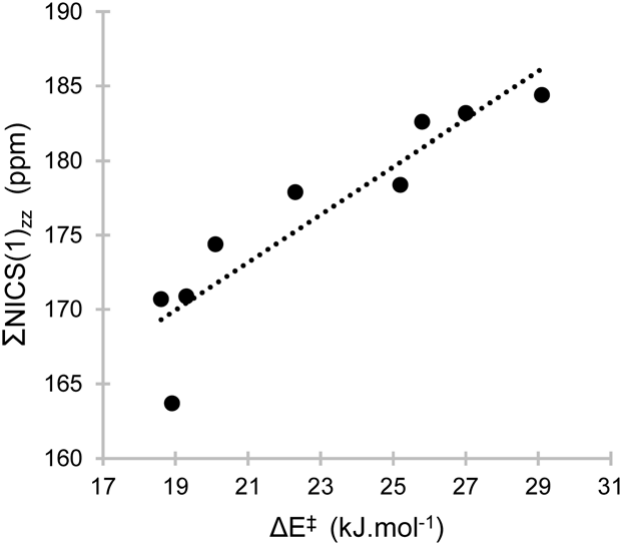
Sum of NICS values for the antiaromatic rings, against the threshold energies of 1^β^ systems.

To explore this possibility, we plotted ΣNICS(1)_*zz*_ (the sum of NICS(1)_*zz*_ values for rings a, a′, and b′) against the threshold energies for 1^β^ with all the studied substituents (Fig. 4). This subset was selected due to the larger number of data points, but we note that similar trends were observed for the limited number of systems of 1^α^ and 2, which were analysed separately because of the strong dependency on the substituents' location. These ΣNICS(1)_*zz*_ values not only showed the strongest correlation (*R*^2^ of 0.84), but also a much larger slope compared to the aromatic rings' dependencies (see SI). In essence, the stronger the paratropicity of a reactant's core, the higher the Δ*E*^‡^. This agrees with our rationalisation above, whereby the effect of any substituent is exacerbated at the TS.

These findings suggest that more intense antiaromaticity, while typically associated with destabilisation, can paradoxically slow down the tunnelling (or lower the splitting energy in a coherent regime) by preferentially destabilising the transition state relative to the reactant. Importantly, it has been previously noted that magnetic antiaromaticity does not necessarily imply instability.^72^ This offers a predictive link between local electronic structure and tunnelling kinetics.

## Conclusions

The dinaphtho[2,1-*a* : 1,2-*f*]pentalene (1) and dinaphtho[1,2-*a* : 2,1-*f*]pentalene (2) systems studied here have energy surfaces with degenerate double-well potentials that show a distinctive divergence in their local aromaticity distribution. We demonstrate that the aromatic and antiaromatic character of specific rings can be switched *via* fast tunnelling, establishing a novel case of dynamic aromaticity. This aromatic unsymmetry, along with the correlated reaction rates, can be modulated by functional group substitutions at key positions.

If the systems can be produced in a coherent regime, they are predicted to exhibit energy splittings on the order of gigahertz, placing them among the highest possible Δ*E*_01_ values driven by heavy atom QT (comparable to fast hydrogen tunnelling). This provides an experimental window toward the spectroscopic detection of their heavy atom tunnelling.

The possible nuclear quantum superposition of the coherent state opens the possibility of having an unusual case where some of its rings would be both aromatic and antiaromatic at the same time. This delocalised situation can be, in principle, maintained as long as the molecules do not decohere by a bath, or by trying to measure their position in the double well potential.

These findings suggest that the studied antiaromatic frameworks can serve as functional platforms for the design of π-conjugated systems with tuneable quantum behaviour, with potential applications in molecular quantum technologies.

## Author contributions

S. J. R. S.: conceptualization; methodology; quantum tunnelling calculations; data curation; figure design; writing. J. J. S. F.: quantum tunnelling calculations; data analysis; writing. K. M. P.: aromaticity calculations; figure design; writing. R. G. P.: conceptualization; aromaticity analysis; data interpretation; writing. S. K.: supervision; conceptualization; methodology; quantum tunnelling analysis; writing.

## Conflicts of interest

There are no conflicts to declare.

## Supplementary Material

SC-016-D5SC05717E-s001

## Data Availability

All the geometries and Gaussian output files are available on the ioChem-BD platform for computational chemistry and materials science teams, at the following link: https://iochem-bd.bsc.es/browse/handle/100/471905. The following references were cited in the supplementary information (SI).^4,40,55,56,58–60,73–76^ Supplementary information with the selection of the electronic structure, full tunnelling tables, aromaticity analysis, and examples of input files is available in a pdf file. See DOI: https://doi.org/10.1039/d5sc05717e.
