# Supplementary material for: Aromaticity switching by quantum tunnelling
Source: Chem Sci. 2025 Oct 7;16(45):21386–93. doi: 10.1039/d5sc05717e (PMC12530803; doi:10.1039/d5sc05717e)
Supplement: SC-016-D5SC05717E-s001 [file SC-016-D5SC05717E-s001.pdf]

Supplementary Information (SI) for Chemical Science.
This journal is © The Royal Society of Chemistry 2025

Supporting Information

Aromaticity Switching by Quantum Tunnelling

Sindy Julieth Rodríguez-Sotelo, Juan Julian Santoyo-Flores, Katarzyna Młodzikowska-Pieńko,

Renana Gershoni-Poranne and Sebastian Kozuch

All the geometries and Gaussian output files are available on the ioChem-BD platform for computational chemistry and materials science teams, at the following link:
https://iochem-bd.bsc.es/browse/handle/100/471905

Electronic Structure Method	S2
Tunnelling Tables	S4
Aromaticity Trends	S6
Aromaticity Values and Figures	S7
Aromaticity Input Templates	S11
Example of Polyrate input files	S13

Electronic Structure Method

Several substituent groups were tested at positions α and β for molecules **1** and **2**. A total of 14 systems that exhibited symmetric isothermal reactions characterised by degenerate double-well potential energy surfaces (DWP) were selected. These also fulfilled the requirement of being closed-shell singlets (CSS) in their reactant and transition state (TS). Such requirement was needed to ensure the high accuracy of the DLPNO-CCSD(T1) computations (**1CN**$^{\alpha}$,**1F**$^{\beta}$, **1SH**$^{\beta}$, **1OH**$^{\beta}$, and **2H** were borderline cases in the TS, but the CSS solution configuration still was of lower energy in coupled cluster).

Due to the size of the systems, DLPNO-CCSD(T1)/cc-pVQZ with TightPNO and TightSCF settings was selected to obtain single-point energies, as it offers an optimal compromise between computational efficiency and high-level accuracy. The size of **1** and **2** makes it impossible to compute canonical CCSD(T) with a relevant basis set. For **1** we managed to compute CCSD(T)/cc-pVDZ, not enough for accurate energies, but useful to establish the ground state configuration. This can be tested when the HF ground state is OSS (obtained with uHF) and therefore can be used as reference for uCCSD(T), against the default restricted HF and CCSD(T) for CSS. Using M062X/6-311+g(d) geometries, coupled cluster clearly indicates that CSS is the correct energy surface for the selected systems. For example, **1F**$^{\beta}$ shows a CSS $\Delta E^{‡}$ of 32 kJ·mol^{-1}, against 85 kJ·mol^{-1} for the OSS (see Table S1).

Table S1. Threshold energies ($\Delta E^{‡}$) in kJ·mol^{-1} for the configuration borderline systems using both closed-shell singlet (CSS) and open-shell singlet (OSS) configurations at the (u)CCSD(T)/cc-pVDZ// M06-2X/6-311+G(d) level.

	CSS	OSS
**1CN**$^{\alpha}$	30	83
**1F**$^{\beta}$	32	85
**1SH**$^{\beta}$	29	83
**1OH**$^{\beta}$	35	88
**2H**	35	92

In addition to the previous test, we conducted further analyses to test the validity of our selected methodology against the problems that can be brought by a multireference character and large static correlation. We calculated four diagnostics over the core of the transition state of the borderline cases (systems **1** and **2**), using the cc-PVQZ basis set (except for T_1, which was calculated with cc-PVDZ).
The first was $A_{25\%}$[1] defined by equation 1:

$$A_{\lambda\%} = 100 \times \frac{1}{\lambda}\left(1 - \frac{TAE[X_{\lambda}C]}{TAE[XC]}\right) \quad (1)$$

Where TAE is the molecular total atomization energy, and $X_{\lambda}C$ is a functional with λ% of exact Exchange, thus comparing DFT results from functionals with and without Hartree-Fock contributions. This takes into account that the Hartree-Fock exchange is less accurate for multireference systems. In this case, we used, as recommended in ref. 1, the PBE and PBE0 functionals, making λ = 25, and as an alternative BLYP vs B1LYP, with the same λ. A $A_{\lambda\%}$ value below 0.010 is considered to describe a single-reference system.

The third diagnostic is the traditional T_1 method,[2] where the vector of coefficients $\vec{t_1}$ for the single coupled cluster excitations is used for the diagnostic, according to

$$T_1 = \|t_1\| / \sqrt{N} \quad (2)$$

Where N is the number of correlated electrons. The values of T_1 should be below 0.02 for organic compounds for the safe use of CCSD(T) derived methods, although some outliers are known.[1]

Finally, we tested our molecules with the %TAE[T] method,[3,4] an energy-based diagnostic used to predict the magnitude of the triples perturbative contributions, which is correlated with the amount of static correlation and with the contribution of post-CCSD(T) terms. In our case, we derived the measure from the DLPNO-CCSD and DLPNO-CCSD(T1) energies, according to:

$$\%TAE[T] = 100 \times \left(1 - \frac{TAE[DLPNO-CCSD]}{TAE[DLPNO-CCSD(T1)]}\right) \quad (3)$$

%TAE[T] values smaller than 5% indicate that post-perturbational triples contributions are negligible, and therefore DLPNO-CCSD(T1) can be safely used.

Table S2. Multireference diagnostic values for systems **1** and **2**. All the values were obtained using the cc-pVQZ basis set for all the atoms (except T_1, calculated with cc-pVDZ), along with DFT and DLPNO methods discussed above.

Method	System 1	System 2	Threshold
$A_{25\%}$ [PBE]	0.086	0.087	< 0.10
$A_{25\%}$ [BLYP]	0.067	0.067	< 0.10
T_1	0.013	0.014	< 0.02
%TAE[T]	2.4 %	2.5 %	< 5%

Additionally, complete active space self-consistent field (CASSCF) calculations were performed using a (10,10) active space with Def2-TZVP. For all the systems, the ground state was a singlet, with the main component of the wave function being a CSS with a weight between 87 and 93%. All these further justify the use of the single reference CCSD(T) as a benchmark.

[1] U.R., Fogueri, S. Kozuch, A. Karton, J.M.L., Martin, *Theor. Chem. Acc.*, 2013, **132**, 1-9.

[2] T.J. Lee, P.R. Taylor, *Int. J. Quantum Chem.*, 1989, **36**, 199-207.

[3] A. Karton, S. Daon, J.M.L., Martin, *Chem. Phys. Lett.*, 2011, **510**, 165-178.

[4] A. Karton, *WIREs Computat. Mol. Sci.*, 2016, **6**, 292-310.

Tunnelling Tables

Table S2. Reactant frequencies leading to the reaction in s^{-1} (approximated from the frequency of the reactant normal mode resembling the reaction), and computed splitting energies ΔE_{01} in MHz.[5]

	**Substituents**	ν_R	ΔE_{01}
**1**	**H**	4.8×10^{13}	4×10^{4}
**1**$^{\alpha}$	**SH**	5.1×10^{13}	3×10^{5}
	**F**	4.3×10^{13}	2×10^{5}
	**CN**	5.1×10^{13}	4×10^{4}
**1**$^{\beta}$	**CHO**	5.1×10^{13}	6×10^{4}
	**NO**	4.9×10^{13}	6×10^{4}
	**NO_2**	5.1×10^{13}	5×10^{4}
	**CN**	5.1×10^{13}	5×10^{4}
	**CCH**	4.9×10^{13}	4×10^{4}
	**OH**	5.1×10^{13}	3×10^{4}
	**F**	5.1×10^{13}	3×10^{4}
	**SH**	5.0×10^{13}	2×10^{4}
	**NH_2**	5.0×10^{13}	2×10^{4}
**2**	**H**	5.0×10^{13}	1×10^{4}
	**F**	5.1×10^{13}	3×10^{4}
	**CCH**	5.0×10^{13}	2×10^{4}

[5] S. J. Rodríguez-Sotelo and S. Kozuch, *Chem. Phys. Lett.*, 2025, **864**, 141890.

Table S3. ZPE-corrected threshold energies in kJ·mol^{-1}, imaginary frequencies at the transition state in cm^{-1}, tunnelling rate constants in s^{-1} for three selected temperatures, and transmission coefficients (ratio between total -k- and semi-classical -k_{SC}- rate constants) at 77 K and 298.15 K.

	**Subs.**	$\Delta E^{‡}$	$\nu^{‡}$	**10 K**		**77 K**		**298.15K**		**κ (77 K)**	**κ (298.15 K)**
				k_{QT}	k_{SC}	k_{QT}	k_{SC}	k_{QT}	k_{SC}		
**1**	**H**	23.1	1946	2×10^{9}	10^{-110}	5×10^{9}	4×10^{-4}	5×10^{10}	5×10^{8}	1×10^{13}	101.5
**1^{α}**	**SH**	5.2	853	1×10^{11}	10^{-17}	3×10^{11}	4×10^{8}	1×10^{12}	5×10^{11}	6×10^{2}	2.4
	**F**	7.6	1016	6×10^{10}	10^{-29}	2×10^{11}	1×10^{7}	1×10^{12}	3×10^{11}	1×10^{4}	3.9
	**CN**	23.6	2050	1×10^{9}	10^{-112}	4×10^{9}	2×10^{-4}	5×10^{10}	4×10^{8}	2×10^{13}	120.1
**1^{β}**	**CHO**	19.3	1648	3×10^{9}	10^{-90}	1×10^{10}	1×10^{-1}	1×10^{11}	2×10^{9}	9×10^{10}	46.5
	**NO**	18.9	1612	3×10^{9}	10^{-88}	1×10^{10}	3×10^{-1}	9×10^{10}	2×10^{9}	4×10^{10}	40.0
	**NO_2**	18.6	1650	3×10^{9}	10^{-88}	1×10^{10}	2×10^{-1}	9×10^{10}	2×10^{9}	5×10^{10}	40.3
	**CN**	20.1	1753	3×10^{9}	10^{-94}	1×10^{10}	4×10^{-2}	8×10^{10}	2×10^{9}	2×10^{11}	54.0
	**CCH**	22.3	1914	2×10^{9}	10^{-106}	7×10^{9}	1×10^{-3}	7×10^{10}	7×10^{8}	6×10^{12}	96.7
	**OH**	27.0	2471	1×10^{9}	10^{-131}	4×10^{9}	9×10^{-7}	5×10^{10}	2×10^{8}	4×10^{15}	326.2
	**F**	25.8	2198	8×10^{8}	10^{-124}	3×10^{9}	6×10^{-6}	3×10^{10}	2×10^{8}	5×10^{14}	193.3
	**SH**	25.2	1934	5×10^{8}	10^{-121}	2×10^{9}	2×10^{-5}	3×10^{10}	2×10^{8}	1×10^{14}	113.0
	**NH_2**	29.1	2704	5×10^{8}	10^{-141}	2×10^{9}	4×10^{-8}	2×10^{10}	5×10^{7}	5×10^{16}	456.0
**2**	**H**	28.5	2224	2×10^{8}	10^{-138}	6×10^{8}	1×10^{-7}	1×10^{10}	6×10^{7}	6×10^{15}	210.8
	**F**	12.3	1163	1×10^{9}	10^{-53}	6×10^{9}	8×10^{3}	2×10^{11}	4×10^{10}	7×10^{5}	5.1
	**CCH**	21.1	1946	3×10^{8}	10^{-103}	1×10^{9}	3×10^{-3}	5×10^{10}	9×10^{8}	4×10^{11}	53.3

Aromaticity Trends

Figure S1. Correlation between the NICS(1.0)$_{zz}$ values (in ppm) for each ring in **1**$^{\beta}$, and the corresponding activation energies. Trendlines match the colour of each ring in the molecular structure.

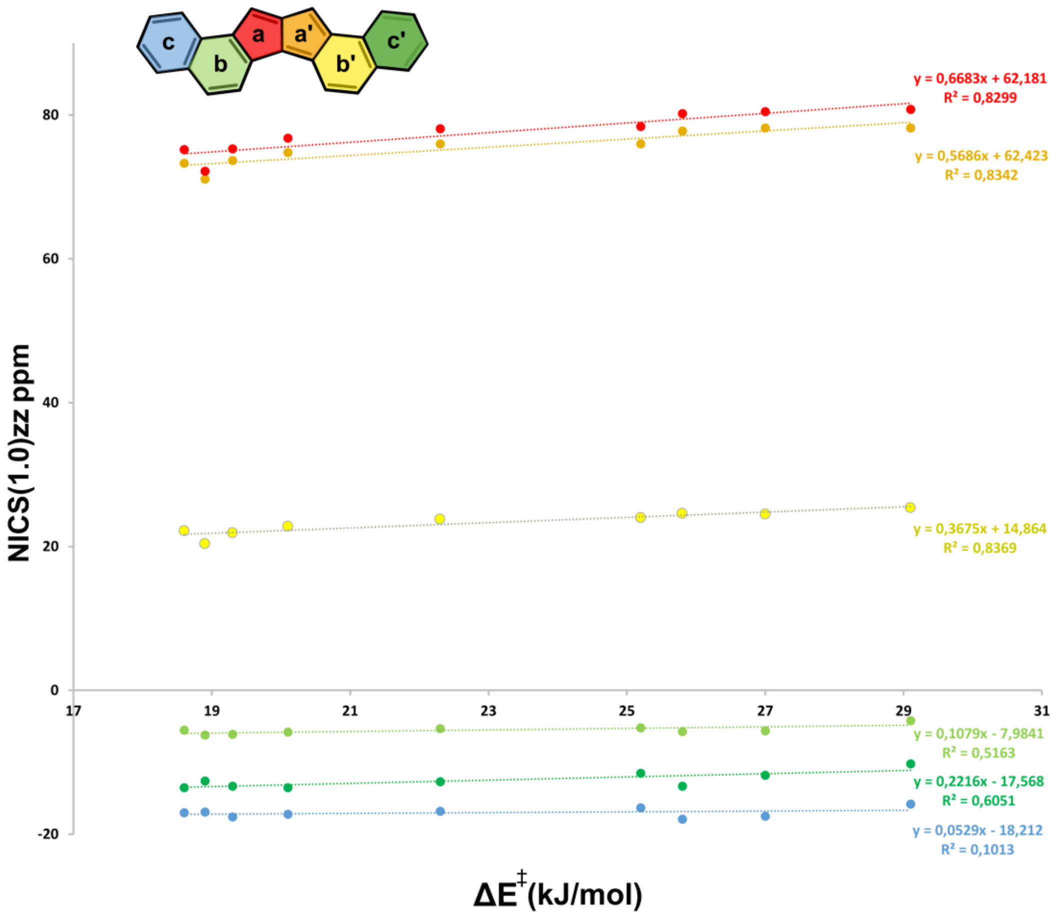

Figure S2. Logarithm of the rate constant at 10 K as a function of the activation energy for **1**.

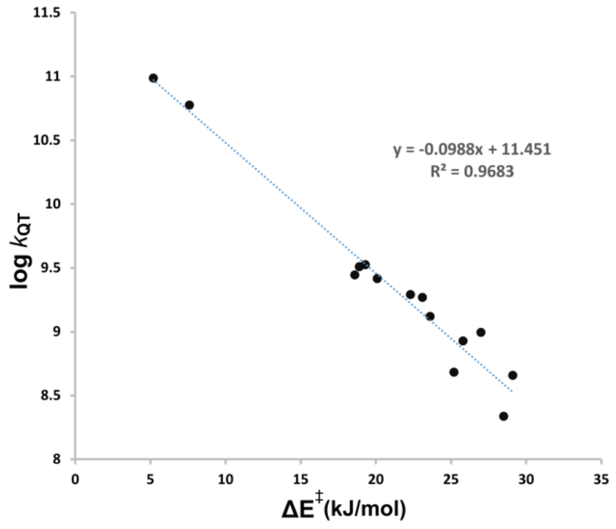

Aromaticity Values and Figures

Table S4. NICS(1.0)$_{zz}$ and NICS(1.7)$_{zz}$ values (ppm) for the |R⟩ state of **1** and **2.**

Sub		NICS(1.0)$_{zz}$						NICS(1.7)$_{zz}$					
		c	b	a	a'	b'	c'	c'	b'	a'	a	b	c
**1**	**H**	-14.2	24.3	79.6	77.6	-5.9	-18.5	-13.1	9.1	32.4	30.1	-7.8	-16.2
**1**[a]	**SH**	-15.6	17.8	71.2	67.8	-6.5	-16.8	-13.9	5.2	27.9	25.3	-7.8	-15.1
	**F**	-15.2	19.3	74.1	70.2	-5.8	-18.6	-13.7	6.2	29.2	26.5	-7.7	-16.2
	**CN**	-10.4	29.2	92.9	84.7	-3.1	-16.2	-11.2	11.7	38.5	33.7	-6.3	-15.0
**1**[b]	**SH**	-11.6	24.0	78.4	76.0	-5.2	-16.2	-11.5	8.9	31.9	29.4	-7.3	-14.7
	**F**	-13.3	24.6	80.2	77.8	-5.7	-17.9	-12.2	9.2	32.7	30.2	-7.7	-15.5
	**CN**	-13.5	22.9	76.8	74.9	-5.8	-17.2	-12.5	8.3	31.0	28.8	-7.6	-15.4
	**CHO**	-12.6	20.4	72.3	71.1	-6.2	-17.0	-12.3	6.9	28.8	27.0	-7.9	-15.4
	**NO**	-13.5	22.2	75.1	73.3	-5.5	-17.0	-12.6	7.9	30.1	28.0	-7.5	-15.3
	**NO_2**	-12.7	23.8	78.1	76.0	-5.3	-16.8	-12.1	8.8	31.7	29.4	-7.4	-15.1
	**CCH**	-11.8	24.5	80.4	78.2	-5.6	-17.5	-11.6	9.1	32.9	30.4	-7.7	-15.3
	**OH**	-13.3	21.9	75.3	73.8	-6.1	-17.6	-12.7	7.7	30.3	28.3	-7.9	-15.9
	**NH_2**	-10.5	25.3	80.7	78.1	-4.2	-15.5	-11.0	9.6	33.0	30.5	-6.9	-14.3
**2**	**H**	-13.8	23.1	74.4	74.8	-8.1	-15.7	-12.6	8.2	30.3	29.4	-8.8	-14.4
	**F**	-15.5	17.6	67.2	65.4	-9.0	-17.0	-13.7	5.0	26.2	24.8	-9.5	-15.1
	**CCH**	-13.0	23.4	79.7	73.9	-7.8	-15.6	-12.1	8.5	32.4	29.1	-8.8	-14.4

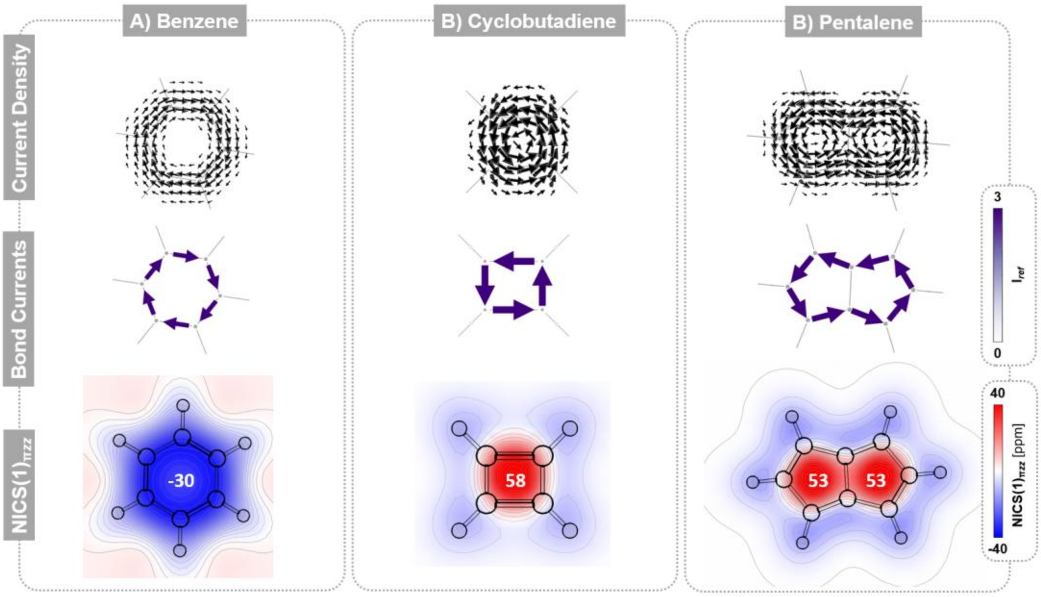

Figure S3. Aromaticity comparison between A) benzene, B) cyclobutadiene, and C) pentalene. (Top) Current density plots generated with SYSMOIC at a height of 1 Å above the molecular plane under a perpendicular magnetic field (only the contributions of the π-orbitals to the induced current density are taken into account). (Middle) Net bond current strengths reported relative to the bond current strength of benzene. (Bottom) 2D NICS(1)$_{zz}$ plots (in ppm). The NICS(1)$_{zz}$ value of each ring is denoted within it.

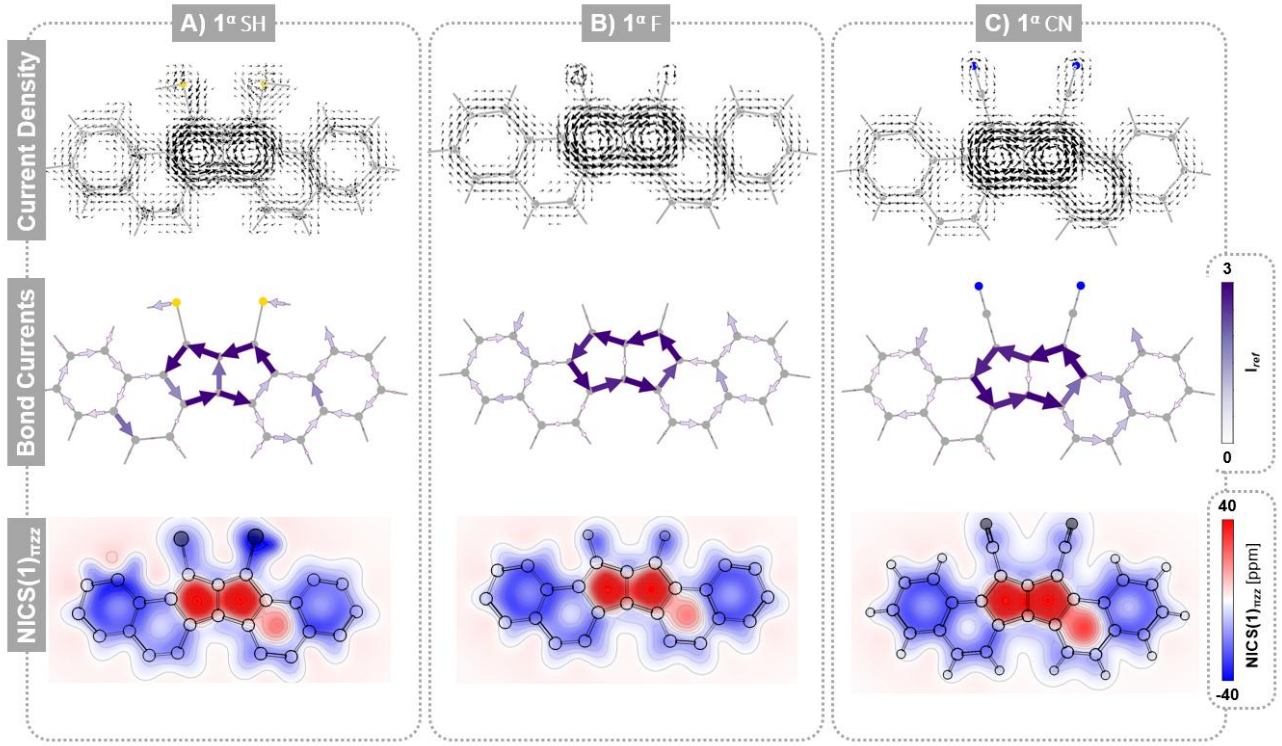

Figure S4. Aromaticity comparison between A) **1SH**$^{\alpha}$, B) **1F**$^{\alpha}$, and C) **1CN**$^{\alpha}$. (Top) Current density plots generated with SYSMOIC at a height of 1 Å above the molecular plane under a perpendicular magnetic field (only the contribution of the π-orbitals to the induced current density are taken into account). (Middle) Net bond current strengths reported relative to the bond current strength of benzene. (Bottom) 2D NICS(1)$_{zz}$ plots (in ppm).

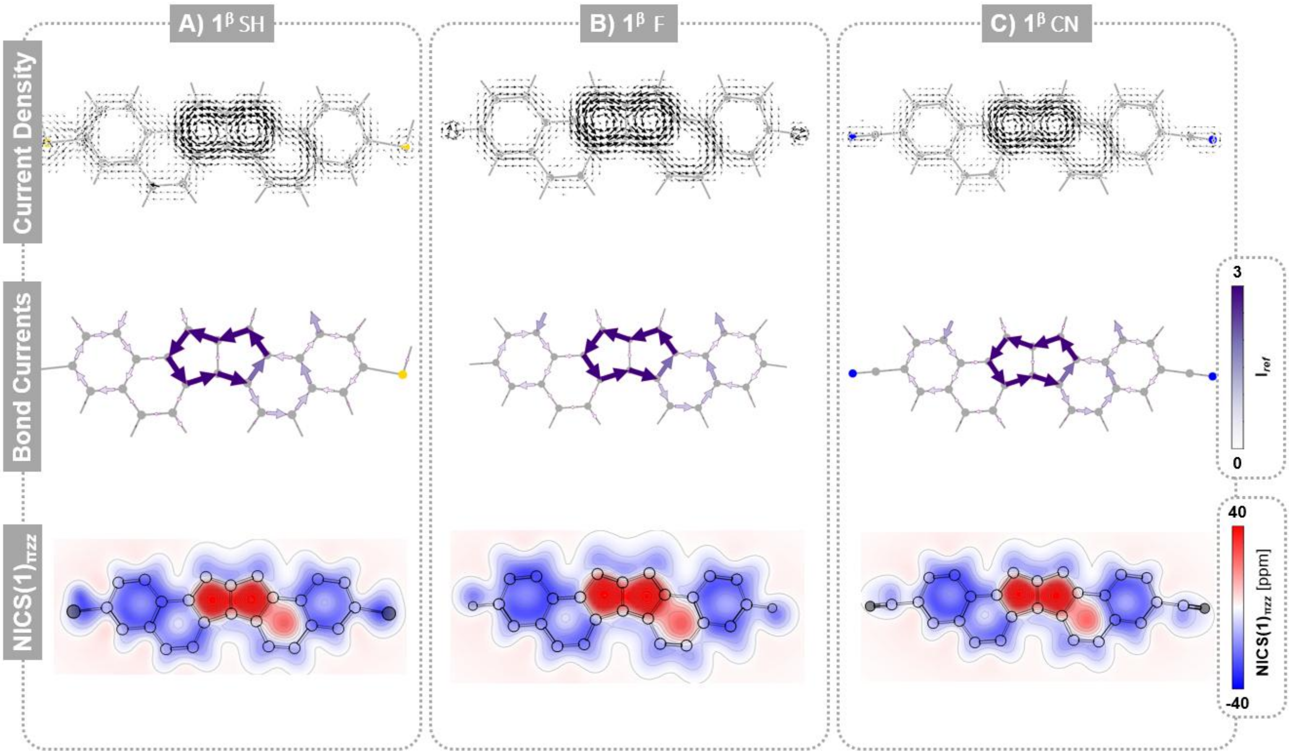

Figure S5. Aromaticity comparison between A) **1SH**$^{\beta}$, B) **1F**$^{\beta}$, and C) **1CN**$^{\beta}$. (Top) Current density plots generated with SYSMOIC at a height of 1 Å above the molecular plane under a perpendicular magnetic field (only the contribution of the π-orbitals to the induced current density are taken into account). (Middle) Net bond current strengths reported relative to the bond current strength of benzene. (Bottom) 2D NICS(1)$_{zz}$ plots (in ppm).

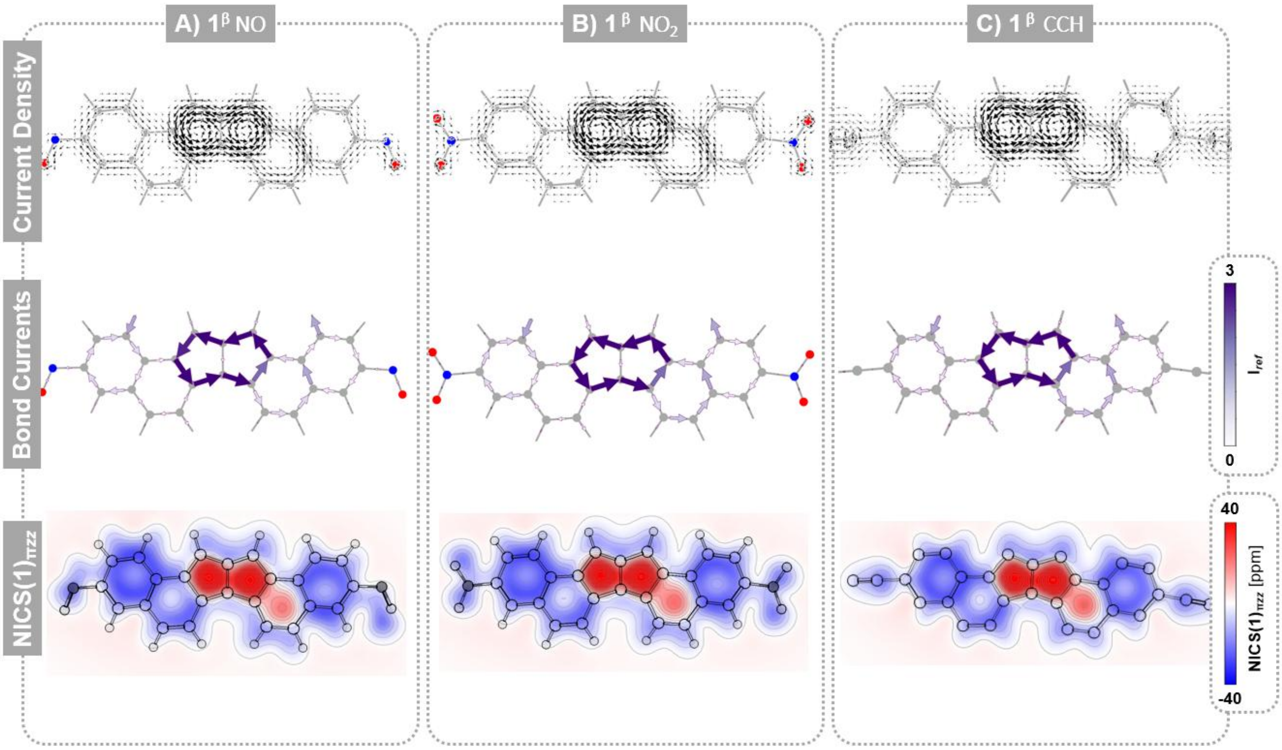

Figure S6. Aromaticity comparison between A) **1NO**$^{\beta}$, B) **1NO$_2$**$^{\beta}$, and C) **1CCH**$^{\beta}$. (Top) Current density plots generated with SYSMOIC at a height of 1 Å above the molecular plane under a perpendicular magnetic field (only the contribution of the π-orbitals to the induced current density are taken into account). (Middle) Net bond current strengths reported relative to the bond current strength of benzene. (Bottom) 2D NICS(1)$_{zz}$ plots (in ppm).

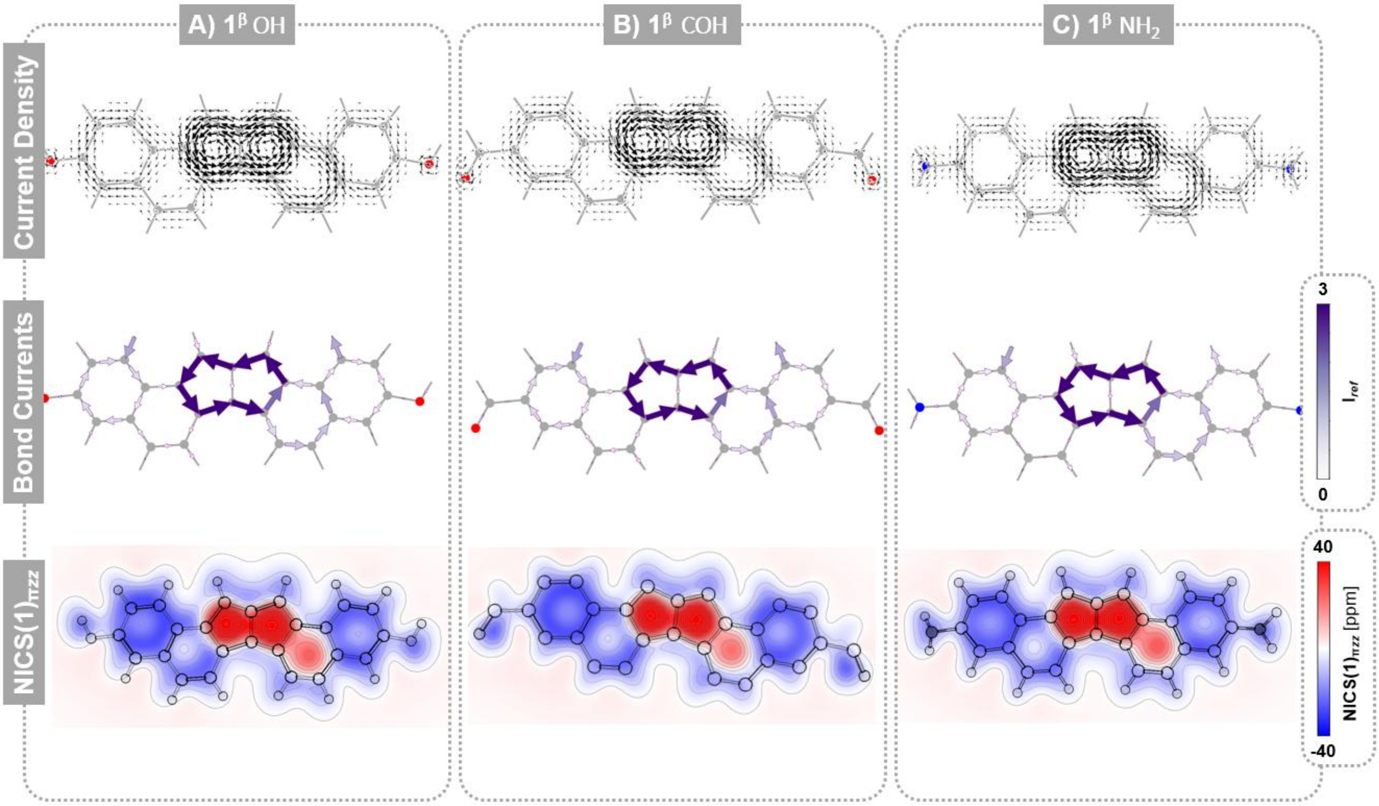

Figure S7. Aromaticity comparison between A) **1OH**$^{\beta}$, B) **1CHO**$^{\beta}$, and C) **1NH$_2$**$^{\beta}$. (Top) Current density plots generated with SYSMOIC at a height of 1 Å above the molecular plane under a perpendicular magnetic field (only the contribution of the π-orbitals to the induced current density are taken into account). (Middle) Net bond current strengths reported relative to the bond current strength of benzene. (Bottom) 2D NICS(1)$_{zz}$ plots (in ppm).

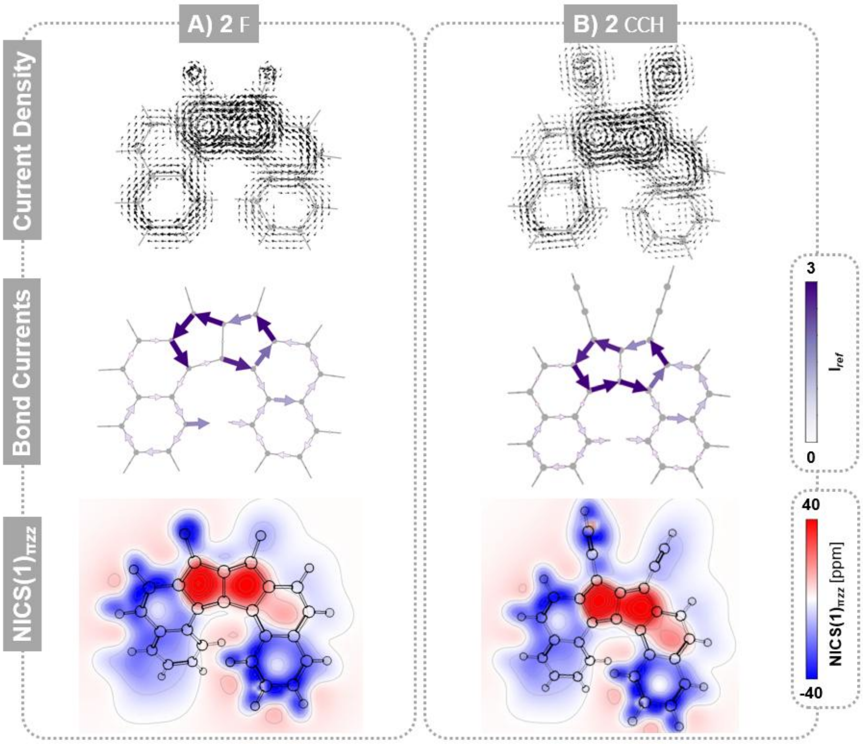

Figure S8. Aromaticity comparison between A) **2F**, and B) **2CCH**. (Top) Current density plots generated with SYSMOIC at a height of 1 Å above the molecular plane under a perpendicular magnetic field (only the contribution of the π-orbitals to the induced current density are taken into account). (Middle) Net bond current strengths reported relative to the bond current strength of benzene. (Bottom) 2D NICS(1)$_{zz}$ plots (in ppm).

Aromaticity Input Templates

NICS(1.0)$_{zz}$ calculated with Gaussian 16[6] (Aroma 2.0[7,8] was used to generate input files)

```
# PBE1PBE/6-311+G(D) NMR=GIAO INTEGRAL=(GRID=ULTRAFINE) CPHF=(GRID=ULTRAFINE)
```

NICS_2D(1.0)$_{zz}$ calculated with Gaussian 16 (pyAroma[9] was used to generate input files)

```
#p nmr=giao PBE1PBE/6-311+G(D) geom=connectivity
```

Current density plots calculated with Gaussian 16 and with SYSMOIC[10,11]

```
#p PBE1PBE/6-311+G(D) output=(wfx,csgtcx) NMR=CSGT
```

Following the Gaussian calculation, the molecular orbitals were visually inspected and the π-orbitals 1Å above the molecular plane were identified (SYSMOIC also does this automatically, but we verified manually).

SYSMOIC calculations to generate the current density plots were performed with the following input format:

```
unpackwfx file
TIPOMO
#current density
JBMAP -qf PIG -o file <jbmap.inp
#current strength
BOCUST -nocd -qf PIG -CS0 11.64 -o file < bocust.inp > file.out
python plot.py compound.3d
```

where file is the basename of the .wfx file (without *.wfx extension) and plot.py is the python code for plotting the current densities presented by Paenurk and Gershoni-Poranne[12]

The jbamp.inp settings were defined as follows:

```
FMM   0.01   0.1
FATT  14.00
STEP  0.60
RI    -18.00 -14.00 -1.9
RF    14.00  15.00  1.9
Y
N
N
```

The RI and RF x- and y-axes were set to be large enough to encompass any compound in the dataset and the z-axis value were set to 1.9 bohr (1.0 Å).

The bocust.inp is a simple text file containing 1500 lines of “y” as the dummy answers to the BOCUST module in order to integrate all the bonds (i.e., the input answers “yes” for every bond BOCUST asks to integrate).

[6] M. J. Frisch, G. W. Trucks, H. B. Schlegel, G. E. Scuseria, M. A. Robb, J. R. Cheeseman, G. Scalmani, V. Barone, G. A. Petersson, H. Nakatsuji, X. Li, M. Caricato, A. V Marenich, J. Bloino, B. G. Janesko, R. Gomperts, B. Mennucci, H. P. Hratchian, J. V Ortiz, A. F. Izmaylov, J. L. Sonnenberg, D. Williams-Young, F. Ding, F. Lipparini, F. Egidi, J. Goings, B. Peng, A. Petrone, T. Henderson, D. Ranasinghe, V. G. Zakrzewski, J. Gao, N. Rega, G. Zheng, W. Liang, M. Hada, M. Ehara, K. Toyota, R. Fukuda, J. Hasegawa, M. Ishida, T. Nakajima, Y. Honda, O. Kitao, H. Nakai, T. Vreven, K. Throssell, J. A. Montgomery, J. E. Peralta, F. Ogliaro, M. J. Bearpark, J. J. Heyd, E. N. Brothers, K. N. Kudin, V. N. Staroverov, T. A. Keith, R. Kobayashi, J. Normand, K. Raghavachari, A. P. Rendell, J. C. Burant, S. S. Iyengar, J. Tomasi, M. Cossi, J. M. Millam, M. Klene, C. Adamo, R. Cammi, J. W. Ochterski, R. L. Martin, K. Morokuma, O. Farkas, J. B. Foresman and D. J. Fox, *Gaussian 16, Revision C. 01*, Wallingford CT, 2016.

[7] A. Stanger, *J. Org. Chem.*, 2010, **75**, 2281–2288.

[8] A. Stanger, *J. Org. Chem.*, 2006, **71**, 883–893.

[9] Z. Wang, *Chemistry*, 2024, **6**, 1692–1703.

[10] P. Lazzeretti and R. Zanasi, *SYSMOIC Package (University of Modena) Additional Routines for Evaluation and Plotting of Current Density by Steiner E. Fowler P., Havenith RWA, and Soncini A.*, 1980.

[11] G. Monaco, F. F. Summa and R. Zanasi, *J. Chem. Inf. Model.*, 2021, **61**, 270–283.

[12] E. Paenurk and R. Gershoni-Poranne, *Phys. Chem. Chem. Phys.*, 2022, **24**, 8631–8644.

Example of Polyrate input files

.dat

```
*GENERAL

TITLE
  Poly_Mol3
END

 DL ISPE

ATOMS
1 C
2 C
3 C
4 C
5 C
6 C
7 C
8 C
9 C
10 C
11 C
12 C
13 C
14 C
15 C
16 C
17 C
18 C
19 C
20 C
21 C
22 C
23 C
24 C
25 H
26 H
27 H
28 H
29 H
30 H
31 H
32 H
33 H
34 H
35 H
36 H
37 H
38 H
END

NOSUPERMOL

*SECOND

HESSCAL hhook

FPRINT

*OPTIMIZATION

PRINT

OPTMIN ohook
OPTTS ohook

*REACT1
INITGEO hooks
GEOM
1
2
3
4
5
6
7
8
9
10
11
12
13
14
15
16
17
18
19
20
21
22
23
24
25
26
27
28
29
30
31
32
33
34
35
36
37
38
END
SPECIES nonlinrp

*PROD1
INITGEO hooks
GEOM
1
2
3
4
5
6
7
8
9
10
11
12
13
14
15
16
17
18
19
20
21
22
23
24
25
26
27
28
29
30
31
32
33
34
35
36
37
38
END
SPECIES nonlinrp

*START
INITGEO hooks
GEOM
1
2
3
4
5
6
7
8
9
10
11
12
13
14
15
16
17
18
19
20
21
22
23
24
25
26
27
28
29
30
31
32
33
34
35
36
37
38
END
SPECIES nonlints
PROJECT

*PATH
#SYMMETRY
INTMU 3
SSTEP 0.001
RPM pagem
SRANGE
  SLP 1.1
  SLM -1.1
END
PRPATH
  coord 1 2
  xmol
  freq 108
END

*TUNNEL
ZCT
SCT
QRST
  harmonic
  mode 108
  states all
END

*RATE
FORWARDK
SIGMAF 1
TST
CVT
PRDELG
PRPART rtp

TEMP
4
5
6
8
10
20
30
40
50
75
77.355
100
125
150
175
194.7
200
225
250
273.15
275
298.15
300
325
350
373.15
375
400
END

ANALYSIS
4
5
6
8
10
20
30
40
50
75
77.355
100
125
150
175
194.7
200
225
250
273.15
275
298.15
300
325
350
373.15
375
400
END

EACT
6. 10.
10. 20.
20. 50.
50. 100.
200. 225.
300. 325.
END

GTLOG
```

.70

```
*GRGENERAL
GRRESTART

*GRSTART
CHARGE 0
MULTIPLICITY 1

*GRCOMMON

GRENER
%mem=100gb
%nproc=32
#n m062x/6-311+g(d)units(au) fchk nosymm
END

GRFIRST
%mem=100gb
%nproc=32
#n m062x/6-311+g(d)units(au) fchk nosymm force
END

GRSEC
%mem=100gb
%nproc=32
#n m062x/6-311+g(d)units(au) fchk nosymm freq
END
```

.71

```
%mem=100gb
%nproc=32
#n m062x/6-311+g(d)fchk nosymm


0 1
C     2.197427   -0.515560    0.000000
C     3.639220   -0.301337    0.000000
C     4.562060   -1.350320    0.000000
C     5.926344   -1.098076    0.000000
C     6.394872    0.215476    0.000000
C     5.490514    1.267094    0.000000
C     4.111934    1.030134    0.000000
C     3.173902    2.150259    0.000000
C     1.834389    1.993330    0.000000
C     1.276607    0.667100    0.000000
C     0.000000    0.196869    0.000000
C    -1.416978    0.577044    0.000000
C    -2.070811    1.815270    0.000000
C    -3.450289    1.844368    0.000000
C    -4.217046    0.656146    0.000000
C    -3.557566   -0.613744    0.000000
C    -4.348975   -1.796274    0.000000
C    -5.714207   -1.723454    0.000000
C    -6.369979   -0.465248    0.000000
C    -5.638445    0.689624    0.000000
C    -2.148865   -0.617494    0.000000
C    -1.201201   -1.755920    0.000000
C     0.057654   -1.256166    0.000000
C     1.463569   -1.659826    0.000000
H     4.204607   -2.374442    0.000000
H     6.627583   -1.924837    0.000000
H     7.460277    0.414579    0.000000
H     5.849576    2.291592    0.000000
H     3.600845    3.148749    0.000000
H     1.175902    2.854466    0.000000
H    -1.505143    2.740096    0.000000
H    -3.971910    2.796171    0.000000
H    -3.857739   -2.763186    0.000000
H    -6.304786   -2.632792    0.000000
H    -7.453233   -0.425834    0.000000
H    -6.134797    1.655086    0.000000
H    -1.489023   -2.799890    0.000000
H     1.833066   -2.675519    0.000000
```

.73

```
%mem=100gb
%nproc=32
#n m062x/6-311+g(d)fchk nosymm

0 1
C    -2.148847   -0.617576    0.000000
C    -3.557566   -0.613842    0.000000
C    -4.348969   -1.796387    0.000000
C    -5.714204   -1.723558    0.000000
C    -6.369977   -0.465353    0.000000
C    -5.638453    0.689533    0.000000
C    -4.217051    0.656035    0.000000
C    -3.450316    1.844286    0.000000
C    -2.070838    1.815200    0.000000
C    -1.416987    0.576975    0.000000
C     0.000000    0.196830    0.000000
C     1.276577    0.667132    0.000000
C     1.834339    1.993368    0.000000
C     3.173856    2.150324    0.000000
C     4.111903    1.030216    0.000000
C     3.639230   -0.301224    0.000000
C     4.562109   -1.350215    0.000000
C     5.926338   -1.097921    0.000000
C     6.394848    0.215670    0.000000
C     5.490501    1.267243    0.000000
C     2.197451   -0.515503    0.000000
C     1.463636   -1.659798    0.000000
C     0.057702   -1.256204    0.000000
C    -1.201149   -1.755970    0.000000
H    -3.857742   -2.763301    0.000000
H    -6.304786   -2.632898    0.000000
H    -7.453233   -0.425963    0.000000
H    -6.134780    1.655008    0.000000
H    -3.971961    2.796073    0.000000
H    -1.505174    2.740027    0.000000
H     1.175860    2.854510    0.000000
H     3.600748    3.148833    0.000000
H     4.204647   -2.374329    0.000000
H     6.627657   -1.924611    0.000000
H     7.460261    0.414708    0.000000
H     5.849469    2.291770    0.000000
H     1.833210   -2.675462    0.000000
H    -1.488975   -2.799939    0.000000
```

.75

```
%mem=100gb
%nproc=32
#n m062x/6-311+g(d)fchk nosymm

0 1
C    -2.183006   -0.563117   -0.000085
C    -3.612734   -0.465466   -0.000106
C    -4.476238   -1.579456   -0.000229
C    -5.843703   -1.411894   -0.000244
C    -6.409994   -0.123967   -0.000137
C    -5.587222    0.978908   -0.000014
C    -4.183761    0.835779    0.000004
C    -3.327619    1.985395    0.000131
C    -1.962344    1.897992    0.000153
C    -1.353406    0.621655    0.000045
C     0.000000    0.192700    0.000032
C     1.353405    0.621655    0.000092
C     1.962344    1.897993    0.000220
C     3.327618    1.985395    0.000245
C     4.183760    0.835779    0.000148
C     3.612734   -0.465466    0.000019
C     4.476239   -1.579455   -0.000073
C     5.843704   -1.411893   -0.000042
C     6.409994   -0.123967    0.000084
C     5.587221    0.978909    0.000177
C     2.183006   -0.563118   -0.000009
C     1.336850   -1.686875   -0.000128
C     0.000000   -1.220645   -0.000103
C    -1.336849   -1.686875   -0.000175
H    -4.056602   -2.579724   -0.000312
H    -6.490930   -2.282052   -0.000339
H    -7.487030   -0.004786   -0.000150
H    -6.010991    1.978563    0.000069
H    -3.800466    2.963399    0.000213
H    -1.356014    2.796409    0.000252
H     1.356014    2.796409    0.000298
H     3.800465    2.963398    0.000343
H     4.056604   -2.579724   -0.000170
H     6.490931   -2.282051   -0.000114
H     7.487031   -0.004784    0.000107
H     6.010990    1.978564    0.000275
H     1.656674   -2.721714   -0.000224
H    -1.656673   -2.721714   -0.000281
```

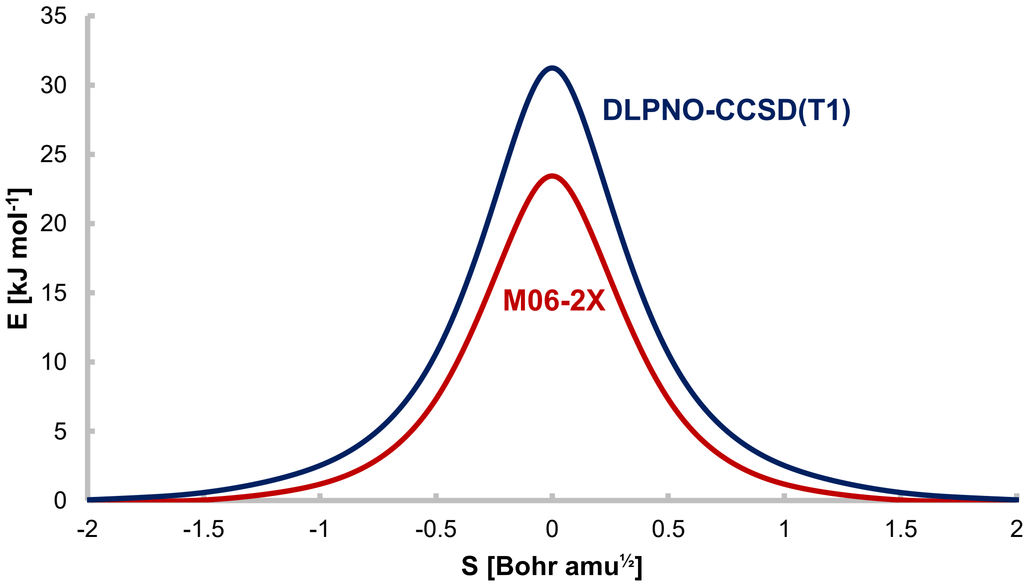

Figure S9. Energy vs. displacement (in mass-scaled coordinates) for **1**, in the DFT PES and with the ISPE correction.
